# Developing Self-Compassion in Healthcare Professionals Utilising a Brief Online Intervention: A Randomised Waitlist Control Trial

**DOI:** 10.3390/ijerph21101346

**Published:** 2024-10-11

**Authors:** Amanda Super, Joanna Yarker, Rachel Lewis, Samuel Keightley, Denvar Summers, Fehmidah Munir

**Affiliations:** 1 Amanda Super Consulting Ltd., Manchester M25 9PH, UK; 2 Birkbeck, University of London, Malet Street, London WC1E 7JL, UK; j.yarker@bbk.ac.uk (J.Y.); rachel.lewis@bbk.ac.uk (R.L.); 3 Affinity Health at Work, 104 Gaskarth Road, London SW12 9NW, UK; 4 King’s College London, Great Maze Pond, London SE1 9RT, UK; samuel.1.keightley@kcl.ac.uk; 5 School of Health & Psychological Sciences, City St. George’s, University of London, Northampton Square, London EC1V 0HB, UK; denvar.summers@city.ac.uk; 6 Work and Health Research Centre, School of Sport, Exercise and Health Sciences, Loughborough University, Spinal Way, Loughborough LE11 3TU, UK; f.munir@lboro.ac.uk

**Keywords:** self-compassion, online intervention, healthcare professionals, workplace, stress management, randomised waitlist control trial

## Abstract

(1) Background: The level of stress experienced by staff in the healthcare sector is highly prevalent and well documented. Self-compassion may support the health and wellbeing of individuals and enable them to stay well at work. This study aimed to understand whether a brief, online, self-guided, novel intervention improved the health and wellbeing of healthcare professionals. (2) Methods: In a parallel randomised controlled trial, a volunteer sample of healthcare professionals were assigned to an intervention group (*n* = 110) or a waitlist control group (*n* = 80). Measures of self-compassion, mental wellbeing, stress and burnout were collected by an online questionnaire at baseline, post-programme and, for the intervention group, at follow-up. (3) Results: This intervention appeared to be effective in increasing self-compassion and mental health and decreasing stress and burnout. Significant group effects and significant time × group interactions for overall self-compassion [F (2, 183) = 32.72, *p* < 0.001; effect size η_p_^2^ = 0.226], mental wellbeing [F (2, 212) = 17.46, *p* < 0.001; effect size η_p_^2^ = 0.135], perceived stress [F (2, 205) = 5.42, *p* = 0.006; effect size η_p_^2^ = 0.46], personal burnout [F (2, 224) = 7.57, *p* = 0.001; effect size η_p_^2^ = 0.063] and work burnout [F (2, 208) = 7.39, *p* = 0.001; effect size η_p_^2^ = 0.062] were found. (4) Conclusions: This study shows promise that an affordable and scalable intervention can be effective for busy healthcare professionals operating in a significantly challenging environment.

## 1. Introduction

In a world beset with volatility, where the future of work is unpredictable and the economic climate precarious, unprecedented levels of workforce stress, anxiety and burnout prevail. Job stress is highly prevalent across the global economy [1], with previous research showing how employees’ health and wellbeing deteriorated during the COVID-19 pandemic [2]. Whilst negatively affecting work performance and job attendance, stress can also lead to psychological health issues such as depression and burnout [3]. Despite formal recommendations [4] to organisations to tackle common mental health issues amongst employees by instigating primary interventions, such as work redesign and organisational change, the United Kingdom’s Health and Safety Executive (HSE) reported 875,000 work-related stress, anxiety and depression cases in 2023. Furthermore, amongst human health and social workers, of the 309,000 estimated cases of work-related ill health, 51% were mental health conditions, a rate which is significantly higher than for other workers across all industries [5].

It is well established that workers in the healthcare sector are particularly vulnerable to stress [6,7] and are most affected by burnout [8], with healthcare professionals requiring support to address the inherent stressors in their work [9]. Crucially, research has shown that high levels of stress in nurses negatively correlates with quality of care provision [10] and may contribute to unsafe practice [11]. With a pressing need to ensure high-quality, safe, effective and compassionate patient care [12], interventions to protect staff and sustain caring behaviours are required [13]. Given the high incidence of stress and burnout in the workplace, self-compassion may be a useful construct to support the health and wellbeing of staff and thus improve patient care. Egan et al. [14] suggest that not applying the development of self-compassion into workplace culture could be detrimental to healthcare workers and represent a form of intra-iatrogenic harm to staff. It is suggested that within healthcare institutions, compassion is essential to promote a culture of care quality and patient safety [15].

Compassion may offer a resource to alleviate the negative effects of workplace challenges and improve personal resources [16,17] and organisations that demonstrate compassion show positive individual and organisational outcomes [18]. Recent reviews and a meta-analysis have shown that mindfulness, meditation and other contemplative interventions, which are increasingly being offered in the workplace to support mental health, are generally affective in reducing employee distress and, in healthcare professionals, stress and burnout [19,20,21,22]. Self-compassion, consisting of aspects of mindfulness, has been recognised as important to health and wellbeing in non-clinical samples over the last fifteen years (see Zessin et al. [23] for a review), with particular relevance to psychological health. In a meta-analysis, Macbeth and Gumley [24] found that self-compassion is a robust predictor of outcomes related to stress and that burnout may also be reduced by self-compassion [25]. Furthermore, studies demonstrate that self-compassion can act as a defending factor against a wide range of wellbeing measures including stress, emotional exhaustion and burnout in healthcare professionals [26,27,28] and can be a useful aid for dealing with everyday worries and anxieties [29].

Self-compassion is understood as compassion directed inward, relating to oneself as the focus of care and consideration when faced with the experience of difficulty [30]. Neff’s approach combines three interrelated components in both the theory and practice of self-compassion: self-kindness as opposed to self-criticism when difficulty is encountered; common humanity, which recognises that all human beings experience challenges as opposed to a sense of isolation and difference to others; and mindfulness, which enables an acknowledgement and acceptance of thoughts and feelings as they occur in the present moment with no judgement, as opposed to reacting and responding to emotion without due insight [30].

However, conceptual and operational overlaps between mindfulness and self-compassion have been identified [31], in that both require approaching difficulty with acceptance, so that reactivity is reduced. Supporting this suggestion, Birnie et al.’s study [32], which employed a community sample, posited that changes in self-compassion were predicted by changes in mindfulness. Similarly, Baer et al. [33] compared the relative predictive utility of self-compassion and mindfulness for psychological wellbeing and found that self-compassion was almost twice as strong a predictor of wellbeing than mindfulness alone, although both were significant predictors. Neff and Dahm [34] argue that as a total construct, self-compassion is broader in scope as self-kindness and common humanity are not qualities that are specifically or inherently part of mindfulness practice [35]. In this, self-compassion encourages the individual to be free from pain and suffering through the act of soothing self-kindness and recognises that challenges are an inherent part of life for all human beings [36]. Meanwhile, mindfulness, in and of itself, regards only the internal experience of the individual to create an increased awareness of thoughts and emotions [37]. The objective of this study was to operationalise a self-compassion-focused development intervention in the workplace and assess self-compassion as a distinct primary outcome variable, whilst mindfulness was considered as a subordinate variable.

A number of randomised controlled studies support the view that a variety of training interventions can improve self-compassion in community samples [36,38]. Recent systematic reviews suggest that self-compassion interventions delivered in the workplace show promise [39,40,41]. However, the reviews highlight the variable quality of studies and, due to a lack of consistency in research design, intervention content and implementation. While there have been recent meta-analyses concerned with self-compassion interventions (e.g., [42,43]), the findings highlight that the quality of studies is limited, and interventions rarely focus on working populations. Given the increasing interest in self-compassion at work, particularly within healthcare settings, there is a need to understand whether the benefits transfer.

Participants in previous intervention studies have shown an increase in self-compassion through learning mindfulness on predominantly Mindfulness-Based Stress Reduction (MBSR) and Mindfulness-Based Cognitive Therapy (MBCT) programmes [9,44]; however, when self-compassion is the explicit focus during an intervention, the effect sizes increase significantly [36]. The Mindful Self-Compassion Programme (MSC) developed by Neff and Germer [36] has been shown to have a number of positive impacts. In their randomised controlled trial, compared to controls, MSC participants demonstrated a significant increase in regard to their self-compassion levels, indicating a large effect size (d = 1.67). Participants also significantly increased their mindfulness, compassion for others and life satisfaction and showed significant decreases in depression, anxiety, stress and emotional avoidance. All significant benefits were maintained at six-month and one-year follow-up. More recently, a study has also shown significant reductions in burnout when employing the MSC intervention with healthcare professionals [45].

Considerations in design and delivery include length and mode of delivery. The MSC intervention consists of 120 to 150 min weekly face-to-face sessions over the course of eight weeks as well as a half-day meditation retreat and 40 min of self-compassion home practice each day. Rees et al. [46] argue that in busy occupational settings, the eight-week duration of conventional interventions (e.g., MSC, MBSR, etc.) may pose a potential barrier in terms of recruitment and retention for participants and limit their broader take-up. They cite evidence of the efficacy of shorter and less intensive mindfulness interventions at work, which have shown significant reductions in burnout symptoms and increased resilience in nurses and healthcare workers (see [47,48,49]). In recognition of the time constraints within a healthcare setting, Neff et al. [50] adapted the MSC and developed the Self-Compassion for Healthcare Communities (SCHC) programme, which consists of six 60 min weekly sessions and, instead of home practice, individuals are encouraged to apply their learning in moments of difficulty at work using key practical exercises. The results showed that this intervention enhanced wellbeing and reduced burnout in healthcare professionals and further small studies considering this approach with similar populations have been positive (e.g., [51]). Furthermore, research has previously shown that even brief self-compassion interventions can impact wellbeing significantly (e.g., [52,53,54]).

Krieger et al. [55] argue that online interventions have many advantages including greater convenience, accessibility and cost-effectiveness as well as removing travel required and affording a higher level of confidentiality than could be provided in a face-to-face group setting, as participants remain anonymous to each other. Online interventions targeting mindfulness have shown promising results in meta-analyses (see [56,57]), while repeated-measure design studies in the field have shown significant improvement following online self-compassion development interventions in a range of populations (see [28,55,58]) and in randomised waitlist control design studies [59,60,61]. Initial findings suggest promise with regard to utilising a non-traditional method of intervention delivery for self-compassion.

This study aimed to examine whether a brief online self-compassion-focused intervention, requiring minimal home practice, to reduce the burden on already-strained individuals, could improve the self-compassion, health and wellbeing of healthcare professionals, and whether these improvements could be maintained across time.

We hypothesised that the primary outcome of self-compassion would significantly improve for the treatment condition when compared to the control group. We also hypothesised improvements in the secondary outcomes of mental wellbeing, stress, personal burnout, work burnout and client-related burnout relative to the control group, and that these improvements would be maintained at one month following the intervention.

## 2. Materials and Methods

### 2.1. Study Design

This parallel randomised controlled trial was conducted with healthcare professionals who received a 4-week online self-guided self-compassion intervention (*n* = 110) compared to a waitlist control group (*n* = 80). Measures were taken at four timepoints: Time 1 (Baseline), Time 2 (post-test for the intervention group), Time 3 (1-month follow-up for the intervention group) and Time 4 (post-test for the control group). An unequal randomisation was performed (see Section 2.9 below). This study is reported according to the CONSORT statement [62] for randomised controlled trials for social and psychological interventions and the TIDieR checklist [63] for reporting intervention descriptions. The trial was not registered prospectively because our primary outcome focused on a psychological mechanism, self-compassion, rather than on a health outcome.

### 2.2. Ethical Statement

Ethical approval was provided for this research, granted by Kingston University, London, and it was conducted in accordance with The Code of Human Research Ethics (2014) [64], as outlined by The British Psychological Society. Authorisation for research to take place was obtained from each participating National Health Service (NHS) trust. This study complied with General Data Protection Regulation (GDPR) restrictions regarding the use of data.

### 2.3. Participants

Healthcare professionals (doctors, nurses, healthcare assistants and other allied health professionals) working in one of five NHS hospital trusts were invited to take part in the study. Study information was circulated to staff using internal e-learning platforms in each of the five NHS trusts: three hospitals, one in the south east of England and two in the north west, and two mental health and community services trusts, one in the south east and one in the north west. Those interested in the study contacted the main researcher via email to obtain a participant information sheet, details of eligibility and a consent form. Eligible participants were contacted once their consent form was received and were asked to complete the study measures via an online questionnaire. Participants were eligible if they were aged over 18 years of age, working a minimum of 30 h per week (as recent research has suggested that reduced working hour arrangements, of less than 30 h per week, are associated with lower allostatic load or chronic stress [65]) had home internet access via a computer/laptop/tablet/smartphone and working Windows Media Player, had not previously received formal training in self-compassion, and were able to commit up to two hours per week for the duration of the four-week intervention study starting in February 2019.

### 2.4. Intervention Group

The intervention consisted of a brief 4-week self-guided online Self-Compassion at Work programme, which was adapted from Mindful Self-compassion (MSC) Programme [36], which the authors describe as being modelled on the structure of Mindfulness-Based Stress Reduction (MBSR) [66]. The Self-Compassion at Work programme also includes elements from other published sources [67,68,69,70,71]. Novel aspects of the intervention are based on the first author’s expertise in the field and an extensive review of the self-compassion development literature (e.g., [37,72,73]), compassionate mind training (e.g., [69]) and compassion development in the workplace (e.g., [74]). The programme employed in this study combines a range of practices derived from the sources stated above and delivers these in a brief, online, self-guided manner, thus accounting for its differing content and delivery method when compared to established compassion development interventions.

The Self-Compassion at Work programme consists of four components, one for each week of the 4-week intervention period. For each week, the intervention participants received the programme via email, which gave programme instructions, the link to the pre-recorded webinar (ranging from 43 to 54 min in duration), a reflective diary and a key task. Each week, participants worked through different practical exercises to develop self-compassion. The programme included watching a recorded webinar with a slide deck that provided individuals with a full grounding in the theory and practice of the three core components of self-compassion, self-kindness, common humanity and mindfulness [30], and practicing breathing and meditation and taking forward concrete exercises to implement at work and in relevant work contexts. Engagement with the programme lasted up to two hours each week and was designed to consider busy work schedules with the practical elements for each of the three core components of self-compassion kept succinct. For example, meditation practices were relatively brief and short informal practice suggestions were presented to incorporate self-compassion into daily habits such as mindful walking, showering and teeth brushing. An action plan was also provided to enable participants to chart their progress throughout the programme. The action plan was attached to the email in week one and in subsequent weeks the email reminded participants to complete it. Participants also received diary-based worksheets each week to promote reflective practice for five minutes each day for the duration of the intervention. A weekly key task of approximately 15 min was provided to embed learning and application relating to the content of each of the webinars (see Table 1 for intervention content). The intervention dosage was carefully considered to ensure all key aspects of the theory and practice of the self-compassion approach were provided, whilst ensuring engagement in the programme was not overly onerous for the participants. None of the documents were shared with the research team as they were for the participants use only, to help embed the ideas presented in the webinars and enhance personal learning. There was no direct contact between the participants and the author, other than to resolve any issues via email, which participants were encouraged to do if they had a query.

To encourage treatment fidelity, at the start of the study, participants were advised that if they completed the programme in full and returned all the questionnaires and the evaluation in the advised timescale, their employing organisation could provide them with a one-day CPD credit.

### 2.5. Control Group

Participants in the waitlist control group were not given any advice or guidance and continued with usual practice until the end of the data collection period. The group were encouraged to complete all three data collection timepoints by being offered the Self-Compassion at Work programme at the end of all data collection. The participants who returned their questionnaires for all data collection timepoints were emailed the programme in its entirety (i.e., links to all four webinars, all tasks, diaries and action plan) in April 2019. Immediately following the four-week intervention period, the waitlist control group were asked to complete a post-programme questionnaire and evaluation.

### 2.6. Piloting of the Intervention

Prior to the main study, the online self-guided Self-Compassion at Work programme was piloted with ten senior healthcare professionals based in the nursing directorate of a large NHS trust from 5 September 2018 to 22 October 2018. All participants completed the eligibility screen and informed consent, pre- and post-evaluation measures and the process evaluation measures. All participants in the pilot study completed the intervention and evaluation measures in full. Five participants took part in semi-structured telephone interviews to provide additional qualitative feedback on both the content and process of the pilot study. The evaluations and feedback from the pilot participants were overwhelmingly positive and they considered the programme both accessible and feasible for a healthcare professional population. However, the pilot provided useful information for minor iterations in relation to the instructions provided on the self-guided Self-Compassion at Work programme, ensuring these were clear before the main trial took place.

### 2.7. Participant’s Personal Measures

Age, gender and place of work were collected by online questionnaire.

### 2.8. Outcome Measures

The following measures were collected from all participants by an online questionnaire at baseline, post-test for the intervention group, 1-month follow-up for the intervention group and post-test for the control group.

#### 2.8.1. Primary Outcome

Self-Compassion: The primary outcome was change in level of self-compassion, measured by the 26-item Self-Compassion Scale (SCS) [75], a valid and reliable self-report measure widely used to assess self-compassion across six subscales: self-kindness, self-Judgement, common humanity, isolation, mindfulness and over-identification. The SCS is rated on a 5-point Likert scale ranging from 1 (almost never) to 5 (almost always), with sample items including, “I try to be understanding and patient towards those aspects of my personality I don’t like” (self-kindness) and “When something painful happens I try to take a balanced view of the situation” (mindfulness). The SCS has adequate construct and convergent validity [75]. As the subscales self-judgement, isolation and over-identification are negatively worded, these were reverse-scored before a total mean score was calculated [75]. Higher overall self-compassion scores indicated higher levels of self-compassion.

#### 2.8.2. Secondary Outcomes

Mental Wellbeing: Measured by the Warwick-Edinburgh Mental Wellbeing Scale (WEMWBS) [76] and comprises 14 positively worded statements such as “I’ve been dealing with problems well” assessed on a five-point Likert scale 1 (none of the time) to 5 (all of the time). The questionnaire scoring ranges from 14 to 70, with higher scores indicating higher mental wellbeing. The WEMWBS has been shown to have good content validity and has near-normal population distribution with no ceiling effects [77].

Stress: Assessed by the 10-item Perceived Stress Scale (PSS) [78], which measures respondents’ sense of control over challenging events and their ability to cope with them. Each item is scored on a 5-point scale with responses ranging from 0 (never) to 4 (very often), with a sample item including “How often have you felt confident about your ability to handle your personal problems?” The scale has indicated good concurrent validity and internal consistency [79]. Lower scores indicated lower levels of stress.

Burnout: Measured using the Copenhagen Burnout Inventory (CBI) [80], a validated self-report 19-item scale which measures personal, work and client-related burnout. The CBI has been found to have high internal reliability [80]. Lower scores indicated lower levels of burnout.

Reliability coefficients (Cronbach’s α) for all primary and secondary outcomes across the four timepoints are presented in Table 2. All measures demonstrate appropriate reliability (α > 0.70) across all timepoints.

### 2.9. Randomisation

Participants were allocated a code so their identifying information was not available to the researchers. Randomisation was performed using blocks of 11 for the intervention group and blocks of 8 for the control group following a similar protocol to Halamova et al. [67]. More participants were randomised into the intervention to account for attrition in completing all 4 weeks of the programme [67]. Allocation of groups to each block was random. Randomisation took place after participants had completed their baseline measures. The lead researcher allocated participants to the intervention and waitlist control using a block randomisation process, blind to the participants’ identifying details. To minimise study contamination, all recruitment was conducted online via email and participants were blinded to group membership with no contact with other participants.

### 2.10. Sample Size

A power calculation was performed using G*Power (version 3.1) for a repeated-measures ANOVA within–between interaction, with a power of 0.95, an alpha level of 0.01, medium effect size f = 0.25 and a repeated-measures correlation coefficient value of 0.1. This suggested a total sample size of 102 participants. To allow for attrition, the sample size was inflated by 54% to account for potential loss to follow-up and non-compliance in data completion. The inflation was based on a similar study [58], and adopted a conservative approach, recognising that as a voluntary intervention, most likely to be engaged with in non-work time, some participants may experience difficulties finding time to engage over the course of the programme [81,82]. To further account for attrition in programme completion in the intervention group, an additional 30 participants were added to this group [67]. This resulted in a total sample size of 188 participants for recruitment (79 participants in the control group and 109 for the intervention group).

### 2.11. Statistical Analyses

All statistical analyses were conducted in SPSS Version 24 [83].

Chi square tests or independent *t*-tests evaluated differences in participant characteristics and the primary outcome measure at baseline. Mixed-model ANOVAs were used to test the difference between the two groups (control and intervention) and difference within each group in outcome measures. Separate repeated-measures ANOVAs were run for the intervention and control group to test differences across the data collection timepoints. Complete data were available for 49% (*n* = 54) of the intervention group and 75% (*n* = 60) of the control group. Data were collected from the control group at T1 (baseline), T2 (post-test for intervention group), T3 (1-month post-test for intervention group) and T4 (post-test for control group). Data were analysed on a complete case basis. For the waitlist control group who received the intervention at the end of the study, paired *t*-tests were run to compare their baseline and post-test measures. In order to interpret effect sizes within intergroup analyses of variance, partial eta squared was presented. For all analyses, the Bonferroni correction was applied at alpha 0.01 to account for multiple comparisons.

## 3. Results

**Figure 1:**
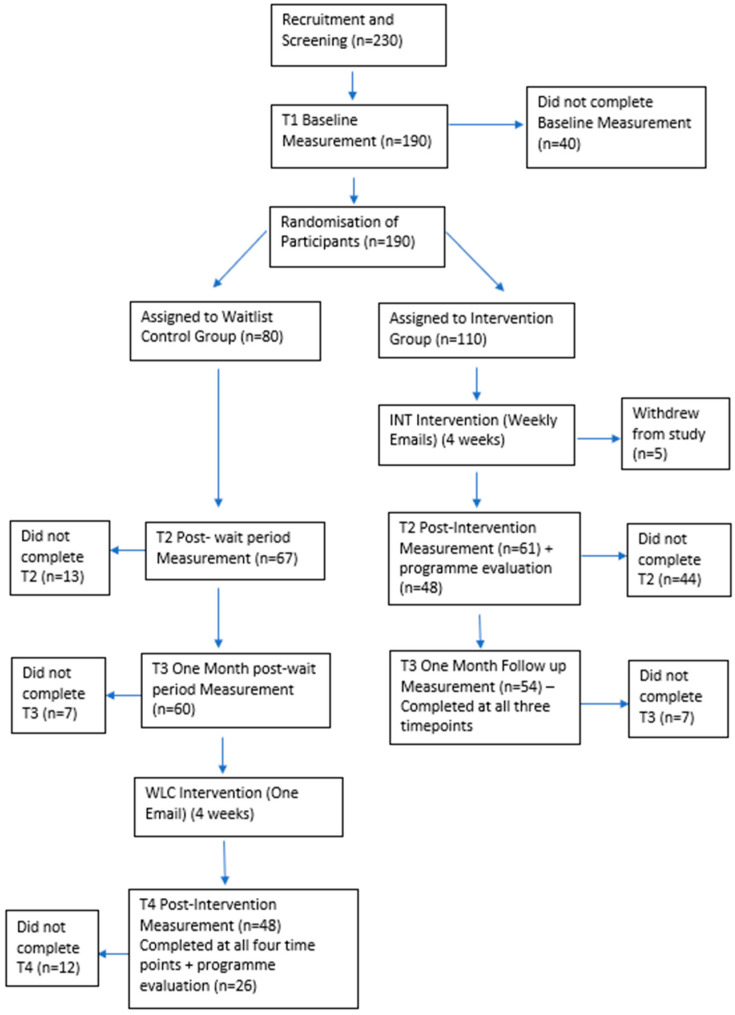
Flow chart to show the number of participants who completed each phase of the study and attrition.

Figure 1 displays the flow of participants through the study with 230 recruited, of which 190 completed the baseline measures and were randomised to the intervention (110 participants) and control group (80 participants) as per the randomisation protocol. Five participants from the intervention group withdrew from the study during the intervention delivery period, citing lack of time due to increased responsibilities at work. Overall, 114 (54%) of the participants remained in the study from baseline to follow-up. More participants in the intervention group withdrew from the study: in the intervention group, 61 (56%) participants completed all measures at T2 (post-test for the intervention group) and 54 (49%) participants at T3 (1-month follow-up for the intervention group). In the waitlist control group, 67 (84%) participants and 60 (75%) participants completed measures at T2 and T3, respectively. Reasons given by both groups for withdrawal were lack of time to take part.

### 3.1. Baseline Characteristics

Table 3 presents the overall characteristics of the intervention and control group. Participant characteristics between the control and intervention group were similar and there were no differences between the groups in self-compassion.

### 3.2. Change in Self-Compassion (Primary Outcome)

As seen in Table 4, there were significant group effects and significant time × group interactions for the overall self-compassion measure [F (2, 183) = 32.72, *p* < 0.001; η_p_^2^ = 0.226], suggesting a positive effect of the online workplace self-compassion programme on the intervention group when compared to the waitlist control group. The repeated-measures ANOVA showed a significant increase in the overall self-compassion scale in the intervention group from baseline to 1-month post-test and to 1-month follow-up (both *p* < 0.001), and no significant change between 1-month post-test and 1-month follow-up assessment (*p* = 0.06), suggesting sustainable effects of the intervention.

For each dimension (subscales) of self-compassion, significant group effects and significant time × group interactions were found: self-kindness [F (2, 211) = 25.69, *p* < 0.001; η_p_^2^ = 0.187], self-judgement [F (2, 191) = 15.71, *p* < 0.001; η_p_^2^ = 0.123], common humanity [F (2, 204) = 14.19, *p* < 0.001; η_p_^2^ = 0.112], isolation [F (2, 185) = 9.76, *p* < 0.001; η_p_^2^ = 0.080], mindfulness [F (2, 224) = 16.59, *p* < 0.001; η_p_^2^ = 0.129] and over-identification [F (2, 199) = 18.14, *p* < 0.001; η_p_^2^ = 0.139]. The repeated-measures ANOVA showed a significant increase in the scores from baseline to 1 month post-test and to 1-month follow-up for self-kindness, common humanity and self-isolation (all *p* < 0.001), but not between 1-month post-test and the 1-month follow-up assessment (*p* = 0.89, *p* = 0.76, *p* = 0.94; respectively).

For the subscales self-judgement, mindfulness and over-identification, a significant effect was found from baseline to 1-month post-test and to the 1-month follow-up assessment (all *p* ≤ 0.001) as well as from 1-month post-test to the 1-month follow-up assessment (*p* = 0.01; *p* = 0.009, *p* = 0.001; respectively), suggesting the effects of the intervention continued to improve scores for these three dimensions of self-compassion after it had been delivered. There were no differences in any of the measures across the data collection timepoints for the control group.

### 3.3. Changes in Mental Wellbeing, Stress and Burnout (Secondary Outcomes)

Table 4 also shows significant group effects and significant time × group interactions for mental wellbeing [F (2, 212) = 17.46, *p* < 0.001; η_p_^2^ = 0.135], perceived stress [F (2, 205) = 5.42, *p* = 0.006; η_p_^2^ = 0.46], personal burnout [F (2, 224) = 7.57, *p* = 0.001; η_p_^2^ = 0.063] and work burnout [F (2, 208) = 7.39, *p* = 0.001], suggesting a positive effect of the self-compassion programme on intervention group when compared to the waitlist control group. There were no significant findings for client-related burnout when applying a Bonferroni correction [F (2, 224) = 4.003, *p* = 0.02; η_p_^2^ = 0.035].

The repeated-measures ANOVA showed a significant increase in mental wellbeing and a significant decrease in perceived stress, personal burnout and work burnout scores in the intervention group from baseline to 1-month post-test and from baseline to the 1-month follow-up assessment (all *p* < 0.01, but not between 1-month post-test to the 1-month follow-up assessment (*p* = 0.430, *p* = 0.198, *p* = 0.223, *p* = 0.395; respectively), suggesting sustainable effects of the intervention. There were no differences in any of the measures across the data collection timepoints for the control group.

### 3.4. Control Group Pre- and Post-Intervention Results

Following availability of the intervention, paired-samples *t*-tests were employed to examine the intervention effects that occurred between the 1-month post-test wait period for the control group (pre-programme) and the final fourth post-intervention measurement (post-programme) for the wait list control group (*n* = 48). As illustrated in Table 5, overall self-compassion, along with the individual dimensions (subscales), displayed a significant positive intervention effect between the two timepoints. All effect sizes from these primary measure analyses ranged from d = 0.5 to 0.81, suggesting the intervention effect was large.

Significant positive intervention effects were also observed among the remaining measures, with significant decreases in perceived stress and burnout measures, whilst mental wellbeing demonstrated a significant increase. Effect sizes for the secondary outcome measures ranged from 0.25 to 0.60, suggesting a varied level of intervention effect.

## 4. Discussion

The aim of this study was to assess the effectiveness of a self-compassion-focused intervention in improving self-compassion and health and wellbeing outcomes among healthcare professionals. The brief online self-guided intervention demonstrated significant positive effects on the primary outcome of self-compassion, and these effects were maintained over time. For the self-compassion subscales, namely self-judgement, mindfulness and over-identification, a significant effect was found not only from baseline to 1-month post-test and to the 1-month follow-up assessment, but also from 1-month post-test to 1-month follow-up, suggesting the effects of the intervention continued after it had been delivered. The intervention also demonstrated significant positive effects on the secondary outcomes of mental wellbeing, stress and burnout. These effects were also maintained over time. Furthermore, the findings support the efficacy of an online, self-guided programme without the need for face-to-face delivery, which often require specialist input and can be difficult to coordinate in healthcare settings [84].

The increased and maintained levels of self-compassion show promise without the need for high intensity or face-to-face provision proposed in previous interventions in work settings [13,36,85]. The findings also contribute to the evidence from previous studies that demonstrate the efficacy of online self-compassion interventions [58,86,87]. The continued increases in three sub-components of self-compassion (self-judgement, mindfulness and over-identification) after the intervention had been delivered suggest that placing a specific focus on self-compassion in the intervention may deliver benefits over interventions that incorporate self-compassion into a wider programme. For example, Duarte and Pinto-Gouveia [88] reported that the self-kindness and self-judgement variables were not significantly affected in their intervention to develop self-compassion using a predominantly Mindfulness-Based Stress Reduction intervention. Echoing a key aspect of the SCHC programme [50], it may be surmised that the Self-Compassion at Work programme provides clear practical exercises (i.e., ‘Self-Compassion Break’ and ‘Three-minute Breathing Space’) that continue to cultivate the three core components of self-compassion. These short practices are easily recalled and can therefore be applied in daily working life on an ongoing basis, which may explain the further increases in, or at least the maintenance of, the beneficial effects seen in this study.

The intervention had a positive effect on mental wellbeing and contributes to the growing evidence on the value of technology-enabled mindfulness-based intervention studies [89,90]. The positive effects were maintained over time, extending the benefits reported in comparable interventions [89]. The intervention provided further support for the role of self-compassion in reducing perceived stress [42,58,86] and the efficacy of the maintained benefits of self-compassion interventions [58]. This is of particular interest within a healthcare environment as high levels of perceived stress have been related to increased absence [91] and reduced care provision [10].

The intervention delivered a reduction in participants’ personal burnout and work-related burnout. The findings add to the body of evidence for Mindfulness-Based Stress Reduction interventions [92,93,94]. This is of particular interest in a healthcare setting due to the well-documented relationship between burnout and compassion fatigue [95] and patient outcomes [96]. No significant effect was found for client burnout. It is possible that the intervention employed in the present study does not have an impact on client-related burnout as operationalised by the Copenhagen Burnout Inventory (CBI) used in this study. However, this scale does not differentiate between ‘regular’ clients and more challenging cases, which could impact stress levels; it is possible that the measure is not sensitive enough to pick such aspects up. It could also be suggested that the organisational processes and structures influence client burnout ratings rather than being affected by the patients (clients) themselves. Mindfulness-based intervention studies that have also employed the CBI measure report no significant effect on burnout [92,93,94]. In contrast, studies that measure burnout using a subscale of the Professional Quality of Life Scale (ProQOL5) [97] have detected changes in burnout following mindfulness-based interventions [13,88] and when employing the Maslach Burnout Inventory (MBI) [98]; following self-compassion-based interventions, changes have been detected in burnout [50], suggesting different sensitivities in these measures.

To understand how wellbeing may be improved in the helping professions, Kinman and Leggetter [99] suggest that nurses need to establish effective emotion regulation approaches, as these are important to enable the renewal of the necessary emotional resources required for the role. In a systematic review conducted by Super [41], a key theoretical underpinning for many of the included intervention studies was based on adaptive emotional regulation, as a means of managing emotion when confronted with stressful events, thus reducing their impact. Gentry and Baranowsky [100] consider that interventions that target adaptive emotional regulation and thought patterns in response to stressors, as well as encouraging alternative responses to work challenges, may be a key feature in developing resilience and decreasing compassion fatigue [46]. Pertinently, Arch et al. [101] proposed that self-compassion training moderates stress responses by enhancing emotional regulation. Recent research has suggested that self-compassion predicts better emotional regulation in relation to both skills and strategies [102] and it appears that self-compassion development, which actively encourages emotions to be present and accepted, may increase psychological strength [103] in the workplace.

There are a number of strengths to this study. The parallel, randomised, controlled trial design, with a large group of healthcare professionals, allowed a direct comparison between the intervention and waitlist control groups, unlike previous studies of self-compassion in the workplace. Although there was a lack of demographical diversity in the present study in relation to gender, with the majority of the participants being female, this characterises the lack of gender diversity in the NHS as a whole. With 80% of NHS staff employed being female [104], a representative sample of the population of interest is provided. The online intervention was designed and delivered to provide assistance to time-poor staff and offer a cost-effective and easy-to-administer format that can target individuals globally, with no direct contact with a mental health professional or waiting lists to negotiate. For a busy working population, a design with less in-person time and no facilitator requirement has advantages over other self-compassion interventions as it can be delivered at low costs and can reach much higher volumes of staff. This is particularly pertinent following the impact of the COVID-19 pandemic, which made face-to-face interventions unlikely to be delivered to healthcare professionals both during and since the lockdowns experienced in the UK. In fact, during the pandemic over 5000 healthcare and other public sector professionals across Great Britain accessed the online Self-Compassion at Work programme, on a complimentary basis, as part of their organisation’s staff health and wellbeing strategy.

Limitations include the attrition of participants and the volunteer sample. While the attrition rate was comparable to other workplace studies [105], it may have been affected by the time of year this study took place, which was a peak period for the healthcare organisations in the UK and the eligibility criteria demanded for this study. The relatively small sample requires replication studies with other healthcare professional samples to corroborate findings. Although no incentives were provided to participants to take part in the study, as a volunteer sample, the participants’ motivation to undertake the intervention may have been higher than in the general healthcare professional population, and therefore may not be entirely representative. Although the generic healthcare professional sample allowed a degree of comparison with previous studies, this study did not consider interdisciplinary differences, which could have provided insights into the impact of the intervention on various roles within the sector. Therefore, future research may consider differences within healthcare organisations such as those who are primarily responsible for providing care to patients and those who are employed in administration and management, as well as considering any differences between professionals based within public and private sectors. Although participants’ initial engagement with the webinar element of the programme was logged, as the virtual software platform (GoTo Webinar Version 10.20.0 Build 19992), commercially employed by the lead researcher, did not provide details of the duration of webinar engagement, the additional programme elements were not requested to be returned and home practice suggestions were not assessed; therefore, treatment fidelity could not be fully established. It is worth noting that the CBI may not have been able to detect sensitivities in relation to client-related burnout in a healthcare professional sample and other measures may offer a more accurate assessment in future studies. An important aspect to acknowledge is that as an individual intervention, the programme employed in this study places the onus on the healthcare professional to develop and benefit from their increased self-compassion alone, with limited impact on the context and system in which they work. Due to time restrictions, the study follow-up was limited to one-month post-programme; future research may therefore consider how the effects of the intervention may be retained across a longer time period. Furthermore, there is no impact suggested by this intervention on the wider organisation, responsible for mitigating and protecting staff from the various risks associated with the high level of challenge experienced in the current NHS. Therefore, employing a framework such as the IGLOO model [106], which suggests that sustainable health and wellbeing is the shared responsibility of the individual, group, leader, organisation and outside community, would be of benefit to inform a system-wide approach. Additionally, the present study lacked an active control condition to allow stronger management of expectancy (placebo) effects, which could indicate that increases may be due to a general treatment effect. Although it has been argued that trials with a waitlist control may overestimate the effects of the treatment condition [107], this can vary depending on study populations. Hence, future studies may look to include an active control group alongside a treatment group and a waitlist control group to enable clear differentiation of the effects on the outcome measures. Similarly, this study did not employ an in-person intervention condition, nor a hybrid in-person intervention with an online self-managed care condition. This should be employed in future research if direct comparisons between intervention types are to be assessed. While the present study is limited in making comparisons with alternative delivery modalities, the efficacy of an online self-compassion development intervention is highlighted.

## 5. Conclusions

This study set out to evaluate a brief online self-compassion development intervention with health professionals using a parallel randomised control design. The findings of this study offer promising evidence to support a brief, self-guided online intervention as an effective and accessible option to increase self-compassion. Furthermore, the intervention led to improvements in mental wellbeing and reductions in stress and burnout. Healthcare professionals operate in a demanding and challenging environment, and it is essential that they are equipped to cope with the inevitable difficulties experienced in the role. Alongside good work design and management, interventions such as this may provide an affordable and scalable approach for organisations looking to enable large numbers of healthcare professionals to stay healthy and well in work.

## Figures and Tables

**Table 1:** The Self-Compassion at Work programme intervention content.

Week/Webinar Title (Time)	Content (Audio Feed and Slide Deck)	Key Task
One—Introduction to the Self-Compassion at Work Programme (43 min)	Welcome to the Programme Facilitator introduction Overview of webinar and practical instructions Introduction to context and need for self-compassion in our lives and in the workplace—the times we live and work in, human cost and impact of the context The benefits that can be derived from developing self-compassion Overview of self-compassion three components with brief practice examples of each: ➣ Clenched fist exercise (self-kindness/self-criticism) ➣ ‘Just like me’ exercise (common humanity/isolation) ➣ ‘Three-minute breathing space’ (mindfulness/over-identification with emotion) An overview of bringing self-compassion to work An outline of the Self-Compassion at Work Programme going forward Reminder of daily diary and week’s key task	Self-Compassion Break Practice to generate the experience of self-compassion (based on [67,68,70])
Two—Introduction to Self-Kindness (45 min)	Overview of the webinar and practical instructions Brief reminder of self-compassion and scientific background Difference between self-compassion and self-esteem Affectionate breathing exercise Our tendency towards self-criticism explained Introduction to self-kindness Self-kindness exercise Developing self-appreciation A self-kindness meditation Physical and emotional self-care suggestions, tips and techniques Reminder of daily diary and week’s key task	Self-Compassion Letter Writing Exercise (based on [67,68,69,70])
Three—Introduction to Common Humanity and Mindfulness (54 min)	Overview of the webinar and practical instructions Introduction to common humanity and connecting with others Loving kindness meditation Expectations, perfectionism and social comparisons Reflective review exercise Cultivating a deeper connection to others with tips and techniques Introduction to mindfulness Research to support mindfulness Mindful moments for stress and anxiety practice exercise Dealing with our emotions and managing difficult feelings Gently exploring feelings in practice Informal daily mindful practice suggestions with tips and techniques Reminder of daily diary and week’s key task	Self-Compassion in Daily Life Exercise (adapted from [67,71])
Four—Developing and Maintaining Self-Compassion Practice (51 min)	Overview of the webinar and practical instructions A recap of the three core components of self-compassion Self-appreciation Meditation on self-acceptance Compassion for others The impact of and reduction in compassion fatigue Giving and receiving compassion meditation Self-compassion and emotional resilience Self-compassion statements exercise Reframing exercise to help in difficult situations Maintaining self-compassion beyond the programme Celebrating our successes Expressing gratitude Guided reflective practice Our self-compassion journey as it continues Reminder of daily diary and week’s key task Thanks for attending and close programme	Gratitude List Exercise (adapted from Appreciation Exercise by [69] and Appreciating Yourself Exercise by [36,67,68])

**Table 2:** Cronbach’s α coefficients for all outcome variables across timepoints.

Measure	Time 1	Time 2	Time 3	Time 4
Overall Self-Compassion	0.94	0.96	0.96	0.94
Self-Kindness	0.87	0.90	0.91	0.90
Self-Judgement	0.82	0.88	0.89	0.86
Common Humanity	0.84	0.87	0.87	0.84
Isolation	0.84	0.85	0.85	0.87
Mindfulness	0.80	0.84	0.85	0.79
Over-Identification	0.81	0.84	0.84	0.70
Mental Wellbeing	0.90	0.93	0.93	0.92
Stress	0.84	0.89	0.89	0.88
Overall Burnout	0.90	0.92	0.92	0.93
Personal Burnout	0.87	0.89	0.91	0.88
Work Burnout	0.78	0.83	0.81	0.82
Client-Related Burnout	0.86	0.90	0.90	0.90

**Table 3:** Baseline characteristics according to randomised groups: waitlist control and intervention.

Variable	Control (*n* = 80)	Intervention (*n* = 110)	Value	*p*
Gender n (%) ^a^				
Male	9 (11.3%)	9 (8.2%)		
Female	71 (88.8%)	100 (90.9%)	4.80	0.48
Age, Mean (SD) ^b^	42.33 (11.06)	41.81 (10.39)	0.329	0.74
Self-compassion (primary outcome) ^b^	Mean (SD)	Mean (SD)		
Self-Compassion (Overall scale)	2.81 (0.76)	2.85 (0.75)	0.230	0.81
Individual self-compassion scales				
Self-Kindness	2.56 (0.91)	2.58 (0.87)	0.329	0.89
Self-Judgement	2.79 (0.91)	2.87 (0.94)	0.230	0.63
Common Humanity	2.72 (0.92)	2.73 (0.96)	0.128	0.95
Isolation	2.93 (1.05)	3.12 (1.03)	0.472	0.34
Mindfulness	3.02 (0.84)	2.92 (0.86)	0.060	0.55
Over-Identified	2.87 (0.98)	2.86 (0.93)	0.954	0.95

^a^ chi-square analyses, ^b^ *t*-tests.

**Table 4:** Mixed-measures ANOVA between time and group waitlist control group (*n* = 60) and intervention group (*n* = 54).

Variable	Baseline Mean (SD)		1 Month Mean (SD)		2 Months Mean (SD)		Group Effect	Time Effect	Time × Group Effect
	Control	Intervention	Control	Intervention	Control	Intervention			
Self-Compassion	2.81 (0.76)	2.85 (0.75)	2.86 (0.75)	3.47 (0.76)	2.86 (0.77)	3.57 (0.72)	F = 11.867	F = 42.249	F = 32.718
							***p* = 0.001**	***p* < 0.001**	***p* < 0.001**
Self-Kindness	2.56 (0.91)	2.58 (0.87)	2.63 (0.85)	3.34 (0.88)	2.53 (0.86)	3.33 (0.89)	F = 11.751	F = 28.444	F = 25.692
							***p* = 0.001**	***p* < 0.001**	***p* < 0.001**
Self-Judgement ^a^	2.79 (0.91)	2.87 (0.94)	2.94 (0.89)	3.54 (0.85)	2.96 (0.95)	3.75 (0.76)	F = 11.030	F = 35.062	F = 15.707
							***p* = 0.001**	***p* < 0.001**	***p* < 0.001**
Common Humanity	2.72 (0.92)	2.73 (0.96)	2.70 (0.99)	3.37 (1.01)	2.81 (0.98)	3.34 (1.03)	F = 6.996	F = 12.730	F = 14.186
							***p* = 0.009**	***p* < 0.001**	***p* < 0.001**
Isolation ^a^	2.93 (1.05)	3.12 (1.03)	3.06 (1.07)	3.79 (0.82)	3.01 (1.04)	3.79 (0.83)	F = 11.805	F = 18.650	F = 9.758
							***p* = 0.001**	***p* < 0.001**	***p* < 0.001**
Mindfulness	3.02 (0.84)	2.92 (0.86)	2.97 (0.79)	3.39 (0.91)	3.00 (0.90)	3.59 (0.87)	F = 4.370	F = 14.528	F = 16.592
							***p* = 0.03**	***p* < 0.001**	***p* < 0.001**
Over-Identification ^a^	2.87 (0.98)	2.86 (0.93)	2.85 (0.99)	3.38 (0.89)	2.94 (0.99)	3.62 (0.90)	F = 5.931	F = 23.693	F = 18.141
							***p* = 0.01**	***p* < 0.001**	***p* < 0.001**
Mental Wellbeing	47.03 (7.56)	46.76 (7.34)	46.47 (7.67)	51.46 (7.38)	46.08 (8.31)	52.04 (7.39)	F = 7.823	F = 9.283	F = 17.459
							***p* = 0.006**	***p* < 0.001**	***p* < 0.001**
Perceived Stress	19.78 (5.68)	19.44 (5.38)	19.10 (5.96)	15.89 (6.31)	19.97 (6.43)	16.78 (6.60)	F = 5.313	F = 9.012	F = 5.421
							***p* = 0.02**	***p* < 0.001**	***p* = 0.006**
Personal Burnout	54.72 (19.38)	53.16 (18.58)	55.35 (20.40)	45.45 (19.14)	55.23 (21.16)	42.67 (19.89)	F = 5.914	F = 5.976	F = 7.567
							***p* = 0.01**	***p* = 0.003**	***p* = 0.001**
Work Burnout	50.36 (16.30)	48.08 (14.60)	51.79 (16.45)	41.13 (17.69)	51.85 (15.83)	42.39 (16.95)	F = 7.389	F = 2.991	F = 7.391
							***p* = 0.008**	*p* = 0.05	***p* = 0.001**
Client-Related Burnout	33.54 (20.68)	34.41 (20.24)	34.03 (21.40)	30.86 (21.83)	35.23 (20.90)	28.01 (20.93)	F = 0.796	F = 1.359	F = 4.003
							*p* = 0.37	*p* = 0.26	*p* = 0.02

^a^ reverse-scored; Bold is signifying statistical significance.

**Table 5:** Means for T3 + T4 measure and paired-samples *t* tests for waitlist control group (*n* = 48) between T3 and T4.

Variable	T3		T4		Paired *t* Tests: T3–T4			
	M	SD	M	SD	*t*	*p*	F95% CI	Cohen’s d
Self-Compassion	2.87	0.76	3.45	0.66	6.34	<0.001	−0.76, −0.40	0.81
Self-Kindness	2.55	0.90	3.25	0.84	5.86	<0.001	−0.96, −0.46	0.80
Self-Judgement	2.95	0.95	3.47	0.82	4.01	<0.001	−0.78, −0.26	0.58
Common Humanity	2.67	1.03	3.43	0.94	5.21	<0.001	−1.04, −0.46	0.77
Isolation	3.03	1.02	3.55	0.96	4.69	<0.001	−0.74, −0.30	0.52
Mindfulness	3.04	0.90	3.46	0.77	3.78	<0.001	−0.65, −0.20	0.50
Over-Identified	2.96	0.92	3.51	0.71	5.51	<0.001	−0.76, −0.35	0.66
WEMWBS	45.46	7.97	50.46	8.56	5.45	<0.001	−6.84, −3.15	0.60
PSS	20.25	6.08	16.73	6.26	3.67	0.001	1.59, 5.45	0.57
CBS								
Personal Burnout	57.47	21.95	47.83	19.71	3.54	0.001	4.16, 15.12	0.46
Work Burnout	53.05	16.83	45.90	17.37	3.60	0.001	3.16, 11.13	0.41
Client-Related Burnout	36.28	21.47	30.82	21.27	2.80	0.007	1.54, 9.39	0.25

Self-Compassion Scale, Warwick-Edinburgh Mental Wellbeing Scale, Perceived Stress Scale, Copenhagen Burnout Index.

## Data Availability

Data used in this publication are available upon request from the corresponding author.
